# Nicole Calakos wins the 2023 ASCI/Stanley J. Korsmeyer Award

**DOI:** 10.1172/JCI169829

**Published:** 2023-04-03

**Authors:** Lisa Conti

The American Society for Clinical Investigation (ASCI) has selected Nicole Calakos as the recipient of the 2023 ASCI/Stanley J. Korsmeyer Award (Figure 1). She is recognized for her contributions to the understanding of brain plasticity and for her discovery of a signaling defect shared by several causes of dystonia. Dr. Calakos is a Professor of Neurology, Neurobiology, and Cell Biology at Duke University Medical Center; the Lincoln Financial Group Distinguished Professor of Neurobiology; and a member of the US National Academy of Medicine. She is a physician-scientist in the field of movement disorders. Recently, Dr. Calakos spoke with the *JCI* about her discoveries and what led to them.

*JCI*: How did you first get interested in science?

Calakos: I remember always loving school, and my enthusiasm for subjects moved around based on teachers who were inspiring to me. I credit the discovery of my passion for science to my high school biology teacher, who suggested I apply to a summer research program for high school students at the University of Florida. Here I was, a 16 year old in a molecular biology laboratory doing work that involved splicing genes. It was really cutting edge and exciting. Once that bug bit, I never turned back and have been really enamored with the creativity and freedom to explore the unknown.

*JCI:* When did you know that you wanted to pursue a career as a physician-scientist?

Calakos: Truthfully, fairly late in college. I was fully immersed in bench research for most of my college time and loved it. My foremost goal was to develop my research interests that were launched in high school. I wanted to go to the University of California, Berkeley, because it was a top ranked research institution and, as a public institution, it was affordable. I am a first-generation college graduate and had to pay for college. In my freshman year, I landed a work study position in Elizabeth Blackburn’s research laboratory to make plasmid DNA preps for lab members. I eventually got the opportunity to develop my own project. The four years that I was in this lab were extremely formative at many levels owing to outstanding mentoring and science. During my time in the Blackburn lab, Carol Greider was a graduate student and sat a few desks away from me. Blackburn and Greider both went on to win the Nobel Prize. Later in my career, as I experienced some of the challenges or inequities of a career in science, I often looked back to this time as a reminder of what a great scientific team looks like. My experience in the Blackburn lab also has strongly informed how I view the importance of basic science. The research in the lab at that time had no translational questions whatsoever; it was oriented at being curious about why there was all this repetitive DNA at the end of chromosomes and how it got there. Fast forward and now we can see just how impactful that work was, as it intersected with many human biology questions. It has shaped how I run my own lab, which is a mix of curiosity-based, basic, or, what I like to call, foundational science, along with some top-down disease-mechanism questions. I think it is important and synergistic to take both approaches.

I was all consumed with bench research as an undergraduate, but around my junior year, I was encouraged to consider medical school, because in high school, I participated in public service activities. And I missed the gratification from those activities. Still, medical school didn’t feel like the right career, considering my passion for research. When I finally learned about MD/PhD programs, I was excited because they had the potential to bring together my two interests.

*JCI:* Did you have an interest in neurology at that point?

Calakos: No. When I applied for an MD/PhD, my undergraduate work was on a single-cell micro-organism and genomic recombination. I chose neuroscience, because I thought it was fascinating and a good topic to study in depth for the period of my PhD training. I think my choice of thesis lab speaks to this fascination I have with wanting to open the black box of the unknown. I joined Richard Scheller’s lab, enticed by the importance of understanding the molecular machinery behind neurotransmission — how synaptic vesicles get to the membrane, how they fuse in the right place, how receptors on the other side of the synapse are lined up. Hardly anything was known! Scheller was exceptionally creative and had the insight to take advantage of the *Torpedo* electric ray to get a purified preparation of synaptic vesicles. It had been a real challenge to purify those small synaptic vesicles away from all the other small vesicles in a cell in order to get the specific molecular components of synaptic vesicles. When I joined, his lab had just cloned the cDNAs for a variety of synaptic vesicle proteins, and new lab members could pick a few to work on. The job was to figure out what they do and what they were important for. I loved it. The lab was full of inspiring and creative people.

*JCI:* How did your early work on the presynaptic nerve terminal lead you to study dystonia?

Calakos: During my neurology residency, I found myself gravitating to questions about how the brain responded to experience — be it through learning, drug exposures, trauma, or strokes — and how resilience, or lack thereof, contributed to my patients’ experiences. This pointed me toward the field of synaptic plasticity. Synaptic plasticity is a major workhorse in the brain’s response to experience. To learn more about this field, I did a postdoc after residency with Rob Malenka, who is a leader in this field. When I started my own lab, my interests were focused on what plasticity does in basal ganglia circuits, because by this time I really enjoyed taking care of people with movement disorders, like Parkinson’s and Tourette’s, and also other rarer movement disorders, like dystonia. The majority of those diseases involve the basal ganglia circuitry to one extent or another. How does this circuitry normally adapt and have plasticity, and when this goes wrong, what diseases occur? By learning more about normal circuitry, can we help those problems?

*JCI:* What was one of your breakthrough points related to your research on dystonia pathogenesis?

Calakos: I always thought I would study synaptic plasticity in dystonia models and come to an understanding through that angle. Somewhat serendipitously, we ended up performing a high-throughput screen that came about because of meeting a single patient in clinic who had a rare genetic variant in the gene that caused a familial form of dystonia, called DYT1. Since this patient didn’t have the DYT1 mutation, but had dystonia, my curiosity was peaked to understand whether this patient’s mutation was related to their dystonia or not. An undergraduate took on this project and found that the protein affected by this single patient’s rare genetic variant was mislocalized in cells, similar to the protein mislocalization that had been reported with the DYT1 mutation. This got us thinking more about the potential that protein mislocalization might be pathologically relevant. At this point, a technician in my lab suggested the potential to create a high-throughput screen. This idea wasn’t part of our lab’s methods, approaches, or focus. It was more of a top-down treatment-oriented approach than bottom-up understanding of the mechanism, like my lab typically did. It was very cool to give an undergraduate and a technician the opportunity to run with their ideas and see that experiment’s impact evolve over time. That beginning led us to mechanistic insights about causes of dystonia and was also the basis for a large drug discovery effort that is now making its way toward human trials.

*JCI:* You’ve contributed to our understanding of brain plasticity and the underlying causes for dystonia. What has surprised you most about your findings?

Calakos: The first surprise was where the high-throughput screen for a modifier of DYT1 dystonia cell pathology led us. I had always thought such a screen would give us insights into treatment options, but it ended up giving us a window into a cause for the disease. Once we dug into the pathway that was genetically modifying the DYT1 cell pathology, we recognized that there were other genetic causes of dystonia that intersected with it. We had convergence of three different forms of dystonia, with genetic evidence supporting a role of this pathway, which regulates protein synthesis. That really focused our interest on this specific pathway. The pathway is known as the integrated stress response, or ISR, because it plays a role in restoring homeostasis after cell stress by regulating protein synthesis initiation through the eIF2α factor.

The next most surprising thing was that the genome-wide screen in HEK cells, which are kidney cells, identified the ISR pathway, which, in the brain, has also been implicated in learning, memory, and synaptic plasticity — my lab’s core interests. This has been an exciting but hard mystery to follow up on. The ISR is such a universal process that responds to cell stress in all cells throughout the body; yet, how is it uniquely used in the brain for learning, memory, and plasticity? Secondly, where is the Achilles heel that explains why disturbing this pathway leads to such a specific clinical problem as dystonia, particularly in its isolated form where you just have the movement problem? In terms of twists and surprises and trying to figure out what it did uniquely in the brain, we developed a reporter that showed us another really unexpected result. The ISR canonically was thought to be just that — a conditional response to stress that would transiently come up in response to a cell stress. It would restore homeostasis and go back down. But we found that in the healthy brain there were some cells that had the pathway activated all the time. This was very unusual and unexpected.

In trying to figure out how it was useful for the ISR to be on all the time in these cells in the normal healthy brain, we discovered that it significantly influenced dopamine and acetylcholine signaling. These two neuromodulators are incredibly important for being permissive for the induction of long-lasting synaptic plasticity in learning and memory. That discovery brought new insights into what the ISR normally does in the brain and how it influences learning and memory as well. This was a basic science discovery that had its impetus in solving a translational question.

*JCI:* What have been some obstacles that you’ve faced and overcome?

Calakos: It takes a lot of time to do a project, and you’re never guaranteed of the outcome. That kind of uncertainty stress and time investment are difficult. I have also been doing science for most of my career when paylines are so tight and competitive that you don’t really have a lot of financial wiggle room for many projects to fail. This has created personal stress, but I also believe it adversely affects the scientific process. Overall though, I’ve been really fortunate to be able to pursue the questions I wanted to pursue, and I have had a lot of freedom.

A second obstacle I am not sure I fully realized until fairly late in my career is that of implicit bias against women as scientists and professionals. I was extremely naive and had not really thought about myself as a woman in science. I just thought of myself as a scientist until pretty far along in my career. After I became faculty, one day I looked up and wondered, where did all the women go? I am here because, despite all the implicit bias that might be weighted to not give women the benefit of the doubt, I continuously have also met people who believed in me and supported me. I also think about others that weren’t so lucky to have the opportunities I did. We need to continue the hard work to address bias at its many levels so we can benefit from all the potential contributors in our society.

*JCI:* How would you describe your approach to mentoring?

I have loved mentoring at Duke because I have the opportunity to work with people across a wide range of experiences and backgrounds. From undergraduates who are just getting their first taste of research at the bench to advanced postdocs and faculty. I especially love being part of the moment when someone first realizes they love science and want to be a scientist.

My mentoring philosophy is to try to create an environment that allows growth for everyone, no matter what their starting place is. I am a big believer that it doesn’t matter where you are now, if you’re excited about growth then we have a common mission. I enjoy the process of mentoring immensely.

## Figures and Tables

**Figure 1:**
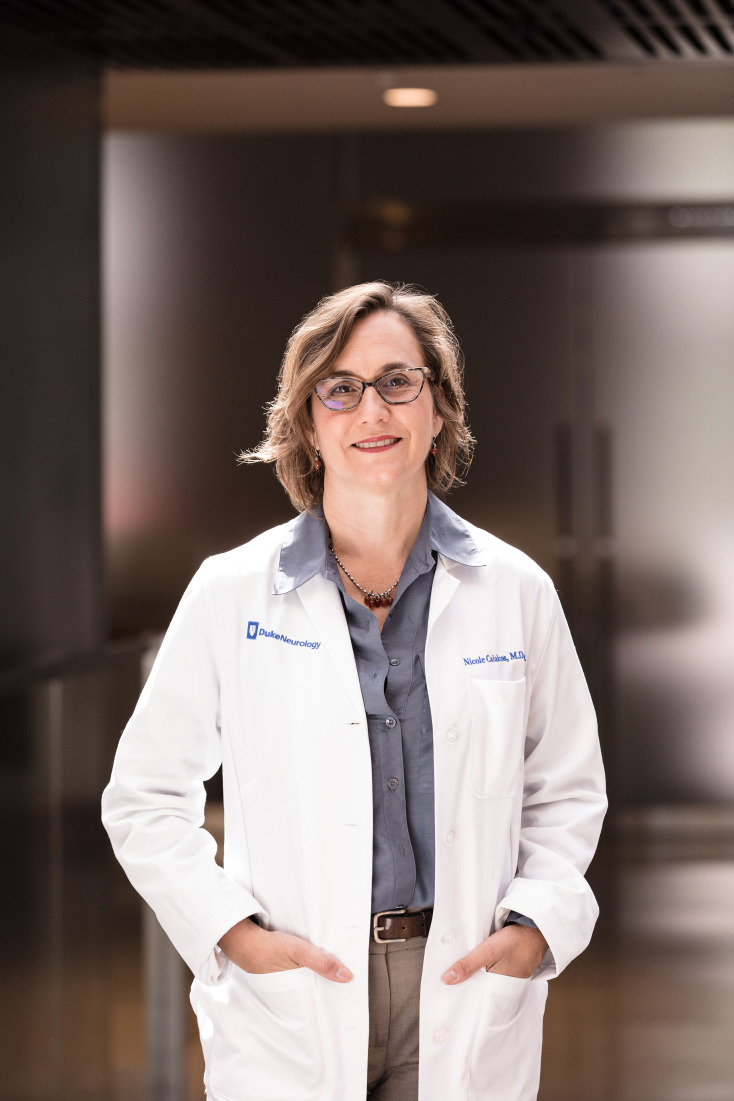
Nicole Calakos is the winner of the 2023 ASCI/Stanley J. Korsmeyer Award. Image credit: Alex Boerner, Duke University School of Medicine.

## Version 1 04/03/2023

Electronic publication

